# Correlates of Lifetime History of Purchasing Sex Services by Men in Saint Petersburg and Leningrad Oblast, Russia

**DOI:** 10.1007/s11524-015-9990-z

**Published:** 2015-10-07

**Authors:** P Girchenko, D. C Ompad, R Kulchynska, D Bikmukhametov, S Dugin, L Gensburg

**Affiliations:** Department of Epidemiology and Biostatistics, School of Public Health, University at Albany, State University of New York, One University Place, Room 131, Rensselaer, NY 12144-3456 USA; New York State International Training and Research Program, New York, NY USA; Global Institute of Public Health, New York University, New York, NY USA; Center for Drug Use and HIV Research, New York University, New York, NY USA; Center for Health, Identity, Behavior, and Prevention Studies, New York University, New York, NY USA; Department of Internal Medicine #1, Dietrich-Bonhoeffer-Klinikum Neubrandenburg, Neubrandenburg, Germany; Fund for Social and Medical Programs Humanitarian Action, Saint Petersburg, Russian Federation

**Keywords:** Men purchasing sex services, Risk of HIV, Sex work, St. Petersburg, Russian Federation

## Abstract

Commercial sex workers (CSWs) in the Russian Federation are at high risk of HIV infection and transmission as a result of unsafe sexual and injecting behaviors. Their clients might be at increased risk of acquiring HIV; however, little is known about the population of men purchasing sex services. This study aims to investigate factors associated with a history of purchasing sex services by men in Saint Petersburg and Leningrad Oblast, Russian Federation. Data were collected as part of a cross-sectional study offering free anonymous rapid HIV testing in Saint Petersburg and Leningrad Oblast in 2014; in total, 3565 men aged 18 years and older provided information about their behaviors associated with risk of acquiring HIV during face-to-face interviews. Prevalence of CSW use in our study was 23.9 %. Multivariable analyses using log-binomial regression were stratified by self-reported HIV testing during the 12 months preceding the study interview. In both strata, older age, multiple sex partners, and a history of sex with an injection drug user (IDU) were associated with an elevated prevalence ratio (PR) for history of purchasing sex services, although the strength of the association differed by strata. Among men who reported recent HIV testing, condom use (PR = 1.22, 90 % confidence interval (CI) 1.0, 1.48) was associated with a history of purchasing sex services, and among men who did not report recent HIV testing, having a consistent sex partner was associated with purchasing sex services (PR = 1.23, 90 % CI 1.1, 1.37). The high prevalence of CSW service use and associations found in this study raise serious concerns about potential for sexual HIV transmission and should be investigated more closely.

## Background

The HIV epidemic in the Russian Federation is one of the fastest-growing HIV epidemics worldwide.^1^^–^3 Injection drug use has been a major driver since the beginning of the HIV epidemic in the Russian Federation,^4^ but during recent years, a major route of HIV transmission has been sexual transmission.^5^^–^7 According to the official statistics from the Federal AIDS Center of Russian Federation, 42 % of 79,728 people diagnosed with HIV in 2013 were infected through heterosexual sex.^8^

With regard to sexual HIV transmission, commercial sex workers (CSWs) represent a population highly vulnerable to HIV infection.^9^^,^^10^ In addition, sex workers in the Russian Federation are at elevated risk of injection drug use,^11^ which places them at higher risk for HIV because of multiple possible routes for HIV acquisition and transmission.^12^^–^14 Consequently, men who use CSW services are at increased risk of HIV infection and represent potential “bridges” for HIV transmission from the vulnerable population of sex workers to the general population.^15^^–^17

Previous research demonstrates that men who pay for sex are likely to have both commercial and non-commercial sex partners, which increase the possibility of HIV transmission to the general population, and places them as an intermediate link between high- and low-risk groups.^17^^–^19 Still, the majority of the studies investigating the impact of sex work on public health, including the role of sexual transmission of HIV infection, have focused on CSWs^20^^–^22 and generally neglected their clients. In many countries including the Russian Federation, male clients of CSWs are not recognized as a separate group at risk of acquiring and transmitting HIV infection by public health officials.^23^

Little is known about male clients of CSW in the Russian Federation, including the prevalence of purchasing sex services, the social and demographic characteristics of clients, and the associations between using CSW services and other behaviors that can increase or decrease risk of HIV infection. The present study’s goal is to examine factors associated with self-reported lifetime experience of using services of commercial sex workers among males in Saint Petersburg and Leningrad Oblast, Russian Federation.

## Methods

### Study Setting

Saint Petersburg is the second largest city in Russia (population 5.1 million), and Leningrad oblast (population 1.7 million) is the region surrounding Saint Petersburg.^24^ Data for the present study were collected as part of free rapid testing for HIV program implemented by the Fund Humanitarian Action^25^ and with administrative support of the state health institution of Leningrad Oblast, the Centre for HIV and Infectious Disease Prevention and Control^26^ during January–December 2014. The behavioral survey was conducted in the frames of pre-testing counseling. Participants were recruited in the public places of Saint Petersburg and Leningrad Oblast, such as subway stations, railroad stations, city squares, malls, and other high population density areas through dissemination of fliers, social advertisement, and word of mouth. A testing van was painted with advertisement of free safe rapid testing for HIV infection, which served as additional attraction for potential participants. Rapid testing for HIV program was implemented by a mobile testing unit of Humanitarian Action and the Centre for HIV and Infectious Diseases Prevention and Control with support of NP E.V.A.,^27^ and funded by Aids Healthcare Foundation (AHF).^28^

### Study Procedures

All individuals who volunteered to undergo rapid testing for HIV were offered an opportunity to complete a brief behavioral survey. Individuals who chose to participate signed a written informed consent form. Data on participants’ demographics and risky behaviors were collected during a face-to-face structured interview. Participation in the survey was completely anonymous; no personal information including names, phone numbers, e-mails, or exact dates of births were collected. Each participant was assigned an individual depersonalized code as a study identifier. Data on socio-demographic characteristics and self-reported risk behaviors were entered on a paper-and-pencil form along with the unique individual code. Data were double entered into an Excel database. Since the behavioral survey and rapid HIV testing were conducted by different organizations, data about HIV status of survey participants are not included in the present study.

### Study Population

The study population consisted of people who volunteered to participate in the survey. Exclusion criteria were age younger than 18 years, visible intoxication or other conditions which would potentially affect the ability to participate, and visible mental disorders.

For the purposes of the current analysis, the sample was restricted to men only. In total, 3682 men were eligible to participate, and 3565 men aged 18–92 gave informed consent and completed the behavioral survey (96.8 % participation rate).

### Statistical Methods

Descriptive statistics were calculated for all variables: means and medians for continuous variables and frequencies and percentages for categorical variables. To compare socio-demographic and behavioral characteristics of the study population to history of purchasing sex services, bivariable analyses were conducted, which included *t* tests for continuous variables and χ^22^ square tests for categorical variables.

Prevalence ratios (PRs) are an appropriate measure of association for cross-sectional studies in which the binary outcomes were common.^29^ Thus, multivariable log-binomial regression, which has been shown to calculate the PR accurately,^30^^,^^31^ was used to measure the associations of interest.

The outcome variable was defined as self-reported lifetime experience of purchasing sexual services from CSWs. The list of potential covariates included variables found to be significantly associated with the outcome of interest in previous research, such as age^20^^,^^32^^,^^33^ categorized into tertiles (29 years and younger (reference group), 30–36 years, and 36 years and older); having a consistent sexual partner^20^^,^^32^^–^34 (yes/no); number of sexual partners during last 12 months^20^ categorized as zero or one (reference group), two to three, and more than four; condom use^35^^,^^36^ during last sex episode; and a history of injection drug use.^34^ Education was not found to be associated with purchasing sex services by the majority of previous studies. However, as education was studied as a potential correlate by other researchers,^20^^,^^33^ it was included in this analysis using categories of less than high school, high school (reference group), some college, and post-college education. In addition, as previous studies found sex work in Russia to be associated with injection drug use,^11^^,^^13^^,^^37^ self-reported injection drug use and history of having sex with an injection drug user (IDU) categorized as yes/no were included in the list of potential covariates. Since previous research does not consider a history of sex with a man and selling sex services as potential covariates of purchasing sex services, it was decided not to include these variables into the models. All covariates except for education and having a consistent sex partner were associated with the outcome in bivariable analyses.

Based on directed acyclic diagram (DAG) analysis, we determined that testing for HIV potentially being a consequence of using services of CSW rather that its predictor might modify the associations between the outcome variable and other covariates. To test this hypothesis, interaction terms of all covariates with recent testing for HIV were included into the full model. Interaction terms between the number of partners during the last 12 months and recent testing for HIV, and also between having IDU as sex partner and recent testing for HIV, were statistically significant (*p* value <0.05), suggesting that associations between sex partners’ characteristics and usage of CSW services are different for those who reported recent testing for HIV and those who did not. Thus, to account for the differences in associations between purchasing CSW services and other covariates for men tested and not tested for HIV during last 12 months, we made a decision to perform stratified analysis. Unadjusted associations between characteristics of the study population and recent testing for HIV were analyzed using χ^22^ tests.

To obtain a parsimonious model, only variables that were significantly associated with the outcome were included in the final models. The purpose of the analysis was exploratory since no other studies were available in the Russian Federation; for this reason, it was decided to use a level of significance of 0.10.

For the two final models (among men with and without HIV testing in the past 12 months), goodness of fit testing was performed using deviance and Hosmer and Lemeshow tests. Data were analyzed using SAS 9.3 software (SAS Institute Inc., Cary, NC, USA).

## Results

### Univariate and Bivariable Analyses

In total, 23.9 % of the study population reported ever purchasing sex services, and only 15.0 % of men participating in the study reported HIV testing during the 12 months preceding the study. Prevalence of injection drug use in the study population was 35.2 %, and prevalence of condom use during the last sexual intercourse was 36.0 %.

Results of bivariable analyses classified by experience of using services of CSWs are presented in Table 1. Men who reported ever purchasing sexual services were older (mean ages 34.5 vs 33.4 years, *p* = 0.008) and more likely to have had multiple sex partners, to have a history of injection drug use, to have had sexual contact with an IDU, and to have had a sex partner of same sex. They were also more likely to use condoms and to be tested for HIV infection. Men with and without a history of purchasing sex services did not differ in terms of education or having a consistent sexual partner, or with regard to experience of selling sex.

**TABLE 1:** Characteristics of the study population by experience of purchasing sex services (*N* = 3565)

Characteristic	No history of purchasing sex services^a^ 2713 (76.1 %)	History of purchasing sex services^a^ 852 (23.9 %)	*p* value
Age category			<0.0001
29 years and younger	1039 (38.3 %)	216 (25.0 %)	
30–36 years	777 (28.7 %)	368 (43.8 %)	
37 years and older	896 (33.0 %)	268 (31.2 %)	
Education			0.653
Unfinished high school	26 (3.0 %)	88 (2.5 %)	
High school	187 (22.0 %)	543 (15.2 %)	
Some college	410 (48.1 %)	1351 (49.9 %)	
Post-college education	229 (26.9 %)	730 (26.9 %)	
Consistent sex partner	1684 (62.2 %)	544 (63.9 %)	0.373
History of injection drug use	903 (33.3 %)	352 (41.3 %)	<0.0001
History of sex with injection drug user	288 (10.6 %)	210 (24.7 %)	<0.0001
History of sex work	14 (0.5 %)	4 (0.5 %)	0.867
History of sex with a man	57 (2.1 %)	36 (4.2 %)	0.0007
Condom use during last sexual intercourse	925 (34.1 %)	359 (42.1 %)	<0.0001
Number of sex partners during last 12 months			<0.0001
1 and fewer	1800 (66.3 %)	311 (36.6 %)	
2–3	144 (5.3 %)	69 (8.1 %)	
4 and more	769 (28.4 %)	472 (55.4 %)	
Tested for HIV during last 12 months	372 (13.7 %)	162 (19.1 %)	0.0002

^a^All values are presented as *n* (%)

Results of bivariable analyses classified by experience of HIV testing are presented in Table 2. There were statistically significant differences by HIV testing status for all characteristics except history of selling sexual services. Men who had an HIV test during the past 12 months were younger (mean age 33.2 vs 33.8 years, *p* < 0.0001), had higher level of education, were less likely to have a consistent sex partner, were more likely to have history of injection drug use and sex with an IDU, and were more likely to report ever having sex with a man. Men who were tested for HIV during the past 12 months were also more likely to report condom use during the last sexual intercourse, have multiple sexual partners, and have history of using services of CSWs.

**TABLE 2:** Characteristics of the study population by HIV testing during the last 12 months (*N* = 3565)

Characteristic	No HIV test during last 12 months^a^ 3031 (85.0 %)	Tested for HIV during last 12 months^a^ 534 (15.0 %)	*p* value
Age category			0.0025
29 years and younger	1077 (35.5 %)	178 (33.3 %)	
30–36 years	940 (31.0 %)	205 (38.4 %)	
37 years and older	1013 (33.4 %)	151 (28.3 %)	
Education			0.0006
Unfinished high school	100 (3.3 %)	14 (2.6 %)	
High school	648 (21.4 %)	82 (15.4 %)	
Some college	1456 (48.1 %)	305 (57.1 %)	
Post-college education	826 (27.2 %)	133 (24.9 %)	
Consistent sex partner	1952 (64.5 %)	276 (51.9 %)	<0.0001
History of injection drug use	990 (32.7 %)	265 (49.6 %)	<0.0001
History of sex with injection drug user	401 (13.2 %)	97 (18.2 %)	<0.0024
History of sex work	16 (0.5 %)	2 (0.4 %)	0.645
History of buying sex services	690 (22.8 %)	162 (30.3 %)	0.0002
History of homosexual sex	71 (2.3 %)	22 (4.1 %)	0.018
Condom use during last sexual intercourse	1068 (35.2 %)	216 (40.4 %)	0.02
Number of sex partners during last 12 months			0.0008
1 and fewer	1834 (60.5 %)	277 (51.9 %)	
2–3	178 (5.9 %)	35 (6.6 %)	
4 and more	1019 (33.6 %)	222 (41.5 %)	

^a^All values are presented as *n* (%)

### Multivariable Analyses

The results of the regression models examining correlates of CSW services differed by HIV testing status during the last 12 months; thus, stratification by HIV testing is justified, and the results are reported separately.

Model 1 (Table 3) demonstrates that, among men not tested for HIV in the past 12 months, the PR of CSW services use for age categories 30 to 36 years and 37 years and older were 1.87 (90 % confidence interval (CI) = 1.63, 2.14) and 1.68 (90 % CI = 1.45, 1.95), respectively, when compared to that for men 29 years of age and younger. In the same model, a history of sexual relationship with IDUs was associated with an increased prevalence of purchasing CSW services (PR = 1.27, 90 % CI = 1.12, 1.43). Having multiple sex partners during the past 12 months had the strongest association with purchasing sex services among men who had two to three sexual partners during the last 12 months (PR = 2.62, 90 % CI = 2.17, 3.17) and among men who had four or more sexual partners (PR = 2.74, 90 % CI = 2.43, 3.10) as compared to having zero to one sex partner during the last 12 months. Having a consistent sex partner was found to be associated with an increased prevalence of purchasing CSW services (PR = 1.23, 90 % CI = 1.10, 1.37).

**TABLE 3:** Prevalence ratios (PRs) and 90 % confidence intervals (CIs) of purchasing sex services by men in Saint Petersburg and Leningrad Oblast controlling for all correlates in the model, stratified by self-reported testing for HIV in the past 12 months

Predictor	Crude PR (90 % CI)	Adjusted PR (90 % CI)
Model 1: Not tested for HIV (*N* = 3029)		
Age category		
29 years and younger	Ref	Ref
30–36 years	1.96 (1.71, 2.25)	1.87 (1.63, 2.14)
37 years and older	1.35 (1.17, 1.58)	1.68 (1.45, 1.95)
History of sex with injection drug user	1.98 (1.76, 2.23)	1.27 (1.12, 1.43)
Consistent sex partner	1.08 (0.96, 1.22)	1.23 (1.10, 1.37)
Number of sex partners during last 12 months		
0–1	Ref	Ref
2–3	2.51 (2.07, 3.05)	2.62 (2.17, 3.17)
4 and more	2.65 (2.35, 2.98)	2.74 (2.43, 3.10)
Model 2: Tested for HIV (*N* = 532)		
Age category		
29 years and younger	Ref	Ref
30–36 years	1.45 (1.11, 1.90)	1.57 (1.22, 2.02)
37 years and older	1.29 (0.96, 1.73)	1.49 (1.14, 1.94)
History of sex with injection drug user	2.01 (1.63. 2.48)	1.73 (1.43, 2.11)
Condom use during last sexual intercourse	1.37 (1.1, 1.69)	1.22 (1.0, 1.48)
Number of sex partners during last 12 months		
0–1	Ref	Ref
2–3	0.97 (0.54, 1.75)	1.03 (0.58, 1.84)
4 and more	2.15 (1.70, 2.70)	2.02 (1.60, 2.55)

Model 2 also found age to be an important correlate of using CSW services among men tested for HIV in the past 12 months; compared to those aged 29 years and younger, those aged 30 to 36 years and 37 years and older were more likely to report use of CSW services (PR = 1.57, 90 % CI = 1.22, 2.02, and PR = 1.49, 90 % CI = 1.14, 1.94, respectively). Similar to men who were not tested for HIV during the past 12 months, experience of sex with IDU was found to be significantly associated with purchasing sexual services (PR = 1.73, 90 % CI = 1.43, 2.11). In contrast to model 1, having two to three sexual partners during the last 12 months was not associated with purchasing CSW services, while having four or more sexual partners during the past 12 months was significantly associated with the outcome (PR = 2.02, 90 % CI = 1.60, 2.55). In model 2, having a consistent sex partner was not significantly associated with history of paying for sex, while condom use during last sexual intercourse was found to be marginally statistically significant.

In both models, age was strongly associated with the outcome, in particular for the age category 30–36 years. The effect of age was stronger among men who had not been recently tested for HIV. Multiple sex partners were also significantly associated with the purchasing of sex services, although for men who did not report recent HIV testing this association was stronger. For men who reported testing for HIV during the last 12 months, the association between history of sex with IDU and history of purchasing CSW services was stronger than for men who did not report recent testing for HIV.

## Discussion

The phenomenon of purchasing sex services in the Russian Federation is under investigation: Sex work is not legal, stigmatized, and not acknowledged as a public health challenge. The purpose of our study was to explore correlates of purchasing sex services by men in Saint Petersburg and Leningrad Oblast. Thus, the study results highlight several potentially problematic issues related to buying sex services and provide avenues for future research. The high prevalence (23.9 %) of using CSW services found in this study, though comparable with prevalence of using CSW services found in other countries,^23^^,^^38^^,^^39^ is striking and requires serious consideration.

Another serious public health finding is the overall high prevalence of injection drug use in the study population. This finding could have resulted from the recruitment procedures, which potentially might have over-sampled populations at high risk for HIV or might truly indicate a high prevalence of injection drug use among men in Saint Petersburg and Leningrad Oblast. According to the Russian Codex of Administrative Offenses against Law, Article 6, Clause 6.9, “Usage of narcotic substances without medical prescription is the subject of financial penalty in the amount of 4000 to 5000 rubles or administrative arrest for up to 15 days.” Because of drug use criminalization, IDUs in the Russian Federation are a hard-to-reach population. The prevalence of injection drug use in the general population is traditionally calculated using the number of people officially registered as injection drug users at public addiction treatment clinics as numerator, which results in significant underestimation in this measure.^40^ Our findings also suggest that prevalence of injection drug use in Saint Petersburg and Leningrad Oblast might be underestimated.

The overall prevalence of not using condoms during last sexual contact was 64 % for the total study population. From a public health point of view, this is a dangerously high proportion. Among those who reported not using condoms, 36.2 % reported having a consistent sex partner and no other sexual contacts, making this a lower-risk group for HIV transmission. The other 64.8 % reported not using condoms and had one or more additional risk factors for HIV transmission, such as multiple sex partners, casual sex partners, or reported usage of CSW services. Of specific concern was the finding that only 42.1 % of the men with a history of CSW service use reported using a condom during their last sexual episode. Thus, the overall level of protective measures taken by men in our study is unacceptably low.

Previous research found testing for HIV to serve as a proxy for perception of the risk of acquiring HIV.^3^ With low rates of HIV testing in our sample, we could suggest insufficient awareness about HIV infection among men. Moreover, we found that among men who reported recent HIV testing, a history of being a CSW client was positively associated with condom use, which provides additional evidence of HIV testing being an index of HIV awareness.

In our study, age was found to be an important correlate of lifetime use of CSW services, which agrees with the results of other studies.^20^ One of the possible explanations for this association is that changes in relationship status, such as divorce, separation, or widowhood resulting in a loss of sex partner, might lead to resolving the problem of sexual satisfaction by using commercial sex services.^20^ Another possible explanation for the association between older age and history of using CSW services is that older age is associated with increased income, making purchasing sex services financially more affordable.^20^

In our study, we observed a strong association between use of CSW services and a history of sex with IDUs. Previous research has demonstrated that in Russia, sex work is associated with the epidemic of injection drug use, placing many CSWs at increased risk for HIV.^12^^,^^13^ The results of our previous study provide some support for the synergism of sex work and injection drug use leading to high rates of HIV infection among CSWs.^37^ In this context, the high prevalence of usage of CSW services by sexually active men of Saint Petersburg and Leningrad oblast, combined with the inadequately low levels of condom use reported by these men, is a serious public health concern.

The finding that, among those men who did not seek testing for HIV during the past 12 months, having a consistent sex partner was associated with a higher prevalence of the purchasing of sex services suggests that, among men with low perception of the risk of acquiring HIV, a stable sexual relationship might not imply monogamy. This statement is supported by the fact that 32.8 % of all men in this study who reported having a consistent sex partner also reported having had multiple partners. Moreover, having a consistent sexual relationship creates a sense of security for both partners and may decrease an already low level of condom use. In our study, men who had consistent sex partner were considerably less likely to use condoms. However, without timely and regular HIV testing, the HIV status of one or both partners in the couple remains unknown. Thus, the sense of security developed in the steady relationship might be false.

These findings raise some serious public health issues for the Russian Federation, including high prevalence of CSW services use, injection drug use, and having multiple sex partners combined with low prevalence of condom use and testing for HIV infection. These risk factors, as well as the associations found in the study, need further exploration and confirmation. However, these data also indicate a need for an immediate public health response including comprehensive HIV prevention programs for sexually active men.

### Strengths

A major strength of this study is the large sample size and the geographical, social, and demographic diversity of the study’s target population. This was achieved through recruitment in a variety of venues, such as malls, railroad stations, and high population density residential areas, allowing the opportunity for participation of different social groups of men.

The method of recruitment, which potentially targeted populations at risk for HIV infection, resulted in the recruitment of a high proportion of men with a self-reported history of purchasing sexual services. In total, 852 men reported to have a history of sex with CSWs, which to our knowledge is the largest sample of CSW clients among all studies conducted in the Russian Federation. Due to the diversity of the study population and the large sample size, associations found in this study are likely to be reliable; however, future studies should confirm these associations and their generalizability in other study populations.

### Limitations

The cross-sectional study design does not allow for assessment of temporality and causality; it is impossible to ascertain if predictors preceded the outcome or vice versa. Nevertheless, for the reasons of exploration and generation of new hypotheses and research questions, the cross-sectional study design was appropriately convenient.

Our study population may not reflect the general population of men in Saint Petersburg and Leningrad oblast, as it potentially may reflect the population of men who consider themselves at higher risk for HIV attracted by the possibility to take a rapid HIV test.

Although the main reason for participation in this study was the incentive of being anonymously tested for HIV infection, the results of the HIV tests were not available for this analysis.

This study is of exploratory nature and was aimed to identify the most urgent issues related to risky behavior for further examination. Thus, the questions did not cover the specific details of risky behaviors. For instance, the survey did not ask more detailed questions about use of CSW services besides ever in lifetime; condom use questions did not ask about specific types of partners. In addition, questions about the different risk factors for acquiring HIV covered different time periods, including lifetime history, the past 12 months, or most recent sexual episode, and we did not have information on all period of interest for certain exposures. In addition, the question about having a consistent sex partner was vaguely worded, and it is unclear what study participants meant when they reported or did not report having a consistent sex partner (e.g., marriage, co-habitation, or regular sex partner).

Potential response bias might have been introduced by the sensitivity of the issues being investigated. Potentially risky behaviors including history of purchasing sex services might be underreported, and protective and socially acceptable behaviors might be over-reported.

## Conclusions

The findings of this study demonstrate the need for an immediate public health response to decrease risk behaviors and increase rates of testing for HIV, particularly among those at high risk. The high prevalence of lifetime CSW services use among the men in our study, though not necessarily reflecting the prevalence of paying for sex among the general population of men in Saint Petersburg and Leningrad oblast, is concerning. Clients of sex workers should be acknowledged as a group at high risk for acquisition and transmission of HIV infection requiring specially tailored HIV prevention and intervention strategies.

Second, the high prevalence of risk behaviors found in our study, such as multiple sex partners and injection drug use, indicates a need for additional research aimed at estimation of prevalence of these types of behaviors in the general population. Low levels of protective behaviors, such as condom use and testing for HIV, indicate insufficient awareness about risk of HIV infection and opportunities for prevention.

Overall, this study indicates a need for urgent public health measures aimed at decreasing risky behaviors and the risk of HIV transmission among men who are clients of CSW, including promoting condom use and regular testing for HIV in both the general population and among men who are clients of CSW.
